# CAN LARGE MULTIMODAL MODELS INTERPRET HEALTH CARE INFOGRAPHICS IN DEMENTIA CAREGIVING MATERIALS?

**DOI:** 10.1093/geroni/igae098.0913

**Published:** 2024-12-31

**Authors:** Zhimeng Luo, Ning Zou, Bo Xie, Daqing He, Robin Hilsabeck, Alyssa Aguirre

**Affiliations:** University of Pittsburgh, Pittsburgh, Pennsylvania, United States; University of Pittsburgh, Pittsburgh, Pennsylvania, United States; The University of Texas Austin, Austin, Texas, United States; University of Pittsburgh, Pittsburgh, Pennsylvania, United States; The University of Texas Health Sciences Center San Antonio, San Antonio, Texas, United States; The University of Texas Austin, Austin, Texas, United States

## Abstract

Infographics are widely used in health education materials for patients and caregivers. As generative artificial intelligence like large multimodal models (LMMs) become increasingly used for content generation, there is a great need to understand if infographics could be used to train LMMs. Addressing such a need, we tested four LMMs, GPT-4V, Gemini-Pro-vision, LLaVA-1.6-34B, and CogVLM-17B, using 5 infographics designed for dementia caregivers as examples. Two independent coders analyzed LMMs’ interpretations of each infographic on accuracy (i.e., the number of model-generated key points that correctly matched those in an infographic divided by the total number of key points contained in the model-generated content) and completeness (i.e., the number of model-generated accurate key points divided by the total number of key points contained in the infographic). Agreement rate was high: Cohen’s kappa=0.8205. Differences were resolved through discussion. Across the 5 infographics, the average accuracy of GPT-4V, Gemini-Pro, LLaVA, and CogVLM was 99.0% (range: 94.4-100%; median: 100%), 83.1% (range: 63.3-100%; median: 81.8%), 76.3% (range: 45.5-100%; median: 81.7%), and 81.5% (range: 50-100%; median: 86.6%), respectively; average completeness was 93.7% (range: 68.8-100%; median: 100%), 53.7% (range: 15.2-77.8%; median: 65.5%), 65.7% (range: 14.3-100%; median: 70.1%), and 37.2% (range: 6.3-77.8%; median: 37.2%), respectively. Overall, GPT-4V significantly outperformed the other LMMs on completeness [F(3,16)=3.55; p=0.04]; no difference was found on accuracy [F(3,16)=2.20; p=0.13]. These findings shed light on LMMs’ abilities to interpret visual and textual data and their applicability in real-world settings like designing high-quality health education materials for patients and caregivers. We conclude with future research directions.

